# Supplementary figures and images for: Combination Effect of Novel Bimetallic Ag-Ni Nanoparticles with Fluconazole against Candida albicans
Source: J Fungi (Basel). 2022 Jul 14;8(7):733. doi: 10.3390/jof8070733 (PMC9316949; doi:10.3390/jof8070733)

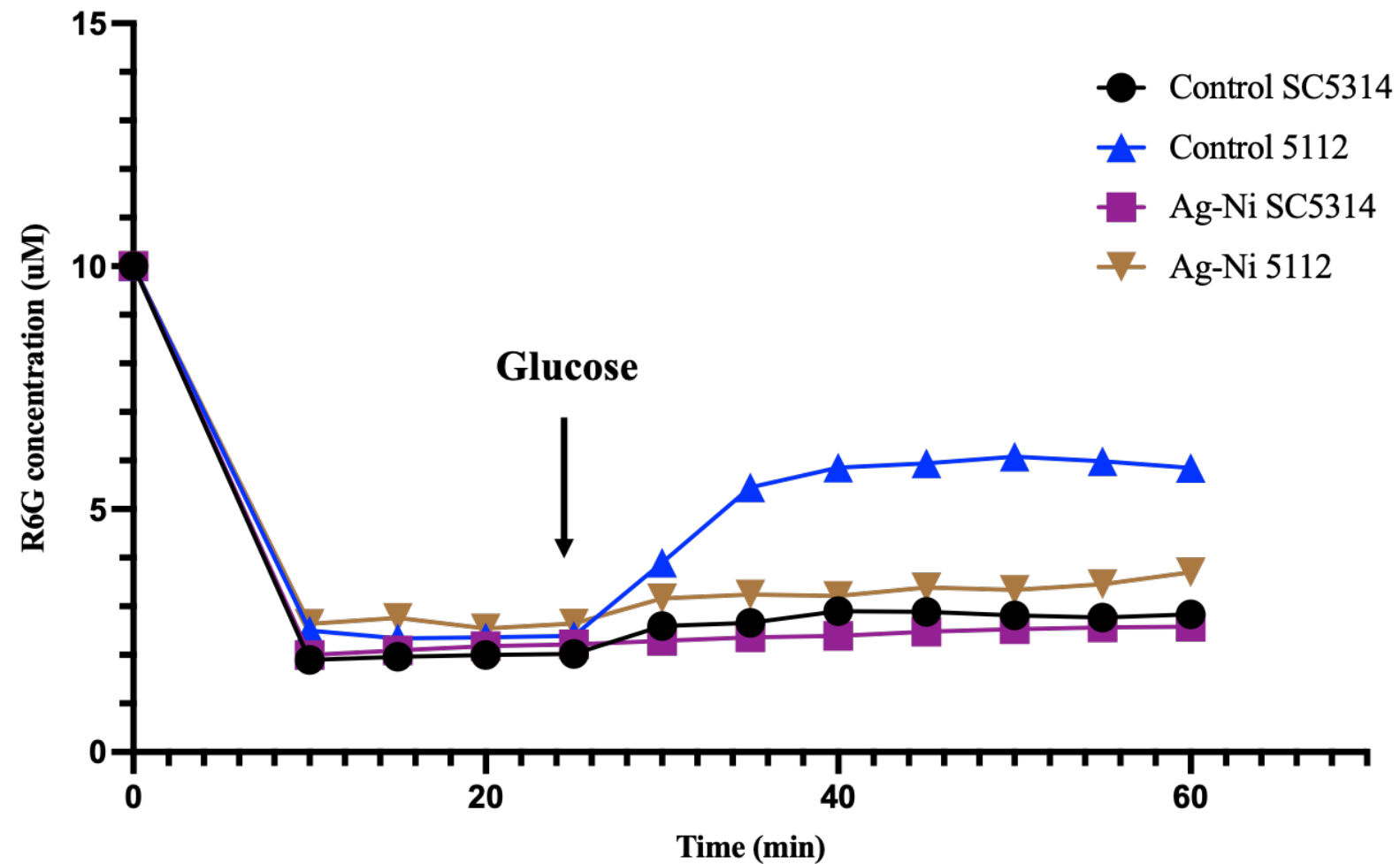

Supplement: Supplementary file 1 [file jof-08-00733-s001.zip › jof-1745432-supplementary.pdf]
